# Proceedings of Workshop on Scientific Writing and Publishing organized by EMAME-DHA at Dubai (March 22-24^th^ 2018)

**DOI:** 10.12669/pjms.344.16084

**Published:** 2018

**Authors:** Shaukat Ali Jawaid

DUBAI: Eastern Mediterranean Association of Medical Editors (EMAME) in collaboration with Dubai Health Authority, Government of Dubai organized a three day Workshop on Scientific Writing and Publishing here from June 22-24^th^ 2018. Mohammad Bin Rashid Academy Medical Center, DHCC, UAE was the venue of this academic activity. Prof. Yousef Al Bastaki Director of Medical Education Dept. in Dubai Health Authority was the moving spirit behind this while others who played a vital role in its organizations included Mr. Ahmad Mandil from WHO EMRO, Dr. Hamid Yahya Hussain Prof. of Community and Family Medicine in DHA, Dr. Waleed Al-Faisal, Prof. Farhad Handjani and Mr. Shuakat Ali Jawaid President and Secretary of EMAME respectively. Other members of the Guest faculty included M. Phillip Dingwall Managing Editor EMHJ from WHO EMRO, Ms. Karen Shashok from Author AID (EMAME) and Phillip Purnell from Knowledge E.

**Figure:**
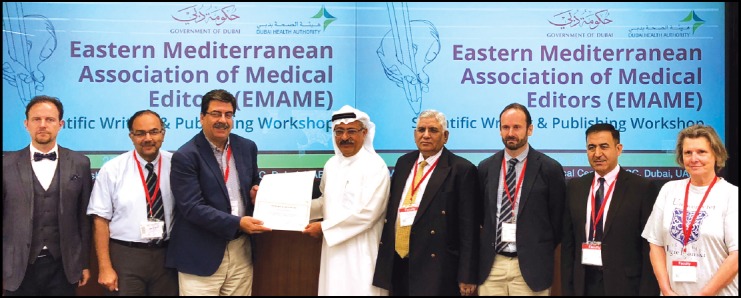
Prof. Farhad Handjani President EMAME alongwith other facilitators of Workshop on Scientific Writing and Publishing presenting a memento to Prof. Yousef Al Bastaki Director of Medical Education Department in Dubai Health Authority who was the moving spirit behind this academic activity.

Speaking in the inaugural session **Prof. Yousef Al Bastaki** introduced the faculty members besides highlighting the aims and objectives stating that it will promote scientific writing skills among young academics and researchers. It will also familiarize the participants with authorship criteria, publication ethics besides submission guidelines. It will also introduce the Journal office set up, rights and responsibilities of Editors. He also gave details of the scientific programme spread over next two and a half days. The expected outcome of this workshop, he stated was to improve the quality of scientific writing by young health professionals which could result in good quality scientific manuscripts being published from the region. It will also increase the understanding of participants of the process involved in submission and publication of manuscripts. They will also gain practical experience in writing scientific articles and have better understanding of ethics of conduct and publishing health research so that they restrain from practices which later on lead to retraction of manuscripts. He also highlighted the importance of indigenous research. English language, he opined, was one of the main problems for the writers and they were also not aware of publication ethics including plagiarism.

**Dr. Ahmad Mandil** from WHO EMRO gave an overview of Scientific Writing and Publishing. A scientific publication, he stated, is a peer reviewed paper published in a scientific journal, peer reviewed book/book chapter. Non-traditional peer reviewed manuscripts include full paper published in proceedings book of a conference or symposium Master or Doctoral Thesis, dissertation, Reports of National Health related agencies like Ministry of Health, Documents or Monographs of health related agencies and NGOs. Structure of a scientific paper consists of Title, Authors, Abstract, Introduction, Methods, Results, Discussion, Acknowledgement and References. The Title, he opined, must be simple, concise, informative, innovative, interesting. It should be considerate of target readership, avoid excessive adjectives, person, place and time of the study.

Title must be simple, concise, informative, innovative, interesting- Ahmed Mandil

Methods should give complete details of the experiment while results should provide answers to questions raised in the introduction. The author should allow different level of readership appreciate and understand important outcome of the study. It should include description of the subjects, response rate, highlight important findings in tables, illustrations. The Discussion should answer research questions. It should also include supportive and non-supportive findings and draw conclusions. It should also state challenges, main findings, highlight shortcomings, compare results with published literature, discuss implications of the findings including possible recommendations. It is important to acknowledge contribution of funding agencies, persons not included in list of authors and contribution of those who helped in collection of data, facilitating of field work etc. He also offered some useful advice to the participants on how to make a presentation, contents of presentation, poster presentation and writing of references.

Methods should cover how the study was designed, how, when and where it was carried out? How data was analyzed?-Shaukat Ali Jawaid

**Prof. Farhad Handjani** talked about Types of scientific articles and mentioned Original articles, Review articles, Case Reports/Case series, and Brief or Short communications, Editorial, Correspondence. Original article produces new knowledge which can include randomized trials, intervention studies, cohort studies, epidemiological assessments, surveys and studies on screening and diagnostic tests. Data should be original and timely and current. Review article is an attempt by researchers to sum-up the current state of research on a particular topic. It usually focuses on advances and discoveries, indicates significant gaps in research in that particular field besides current debates. It can consist of literature reviews or systematic reviews. Review articles usually used to be written by those who are an authority on that subject but these days even the Postgraduates also write reviews. Sometimes it is written by invitation only with special format and set rules. Case reports and Case Series is a description of clinical condition which is not described before. It can be an unusual and reported presentation of some known clinical condition. It can describe unexpected beneficial response to a treatment, description of previously un-reported adverse reactions to treatment. Its format usually requires an abstract or summary and it has a specific writing format. Minoxidil for example was introduced as a drug for hypertension but when it resulted in hair growth, it was used as treatment for hair loss. This finding came from a case report. Brief or Short communication also usually has a structured abstract with limited word count, tables and figures. Correspondence or Letters to the Editor provide supporting information, clarification, criticism, correction or alternative explanations to the results in a previously published article in the journal. It may convey a political, psychosocial message which is related to the practice of medicine and research. Such communications are usually peer reviewed before publication and has a word limitation. Editorial, Prof. Farhad Handjani stated can be a short and pertinent review about a topic which is selected by an Editor. It can be commissioned to an external peer reviewer or expert who can focus commenting on a paper which is being published in the same issue. In Editorial, it is possible to include one’s personal beliefs about that particular topic. It usually has a deadline and it has to be ensured that the author does not have any financial ties to the companies. Other scientific write-ups include Photo Quiz, Medical Hypothesis, Viewpoints, Opinion Corner, Book Reviews, Diagnostic dilemmas, conference reports, poetry, Art of Medicine and obituary notes etc. Details of Tumor Board, Grand Round can also be included in scientific write-ups. He concluded his presentation by quoting Samuel Johnson that “What is written without effort is in general read without pleasure”.

**Figure:**
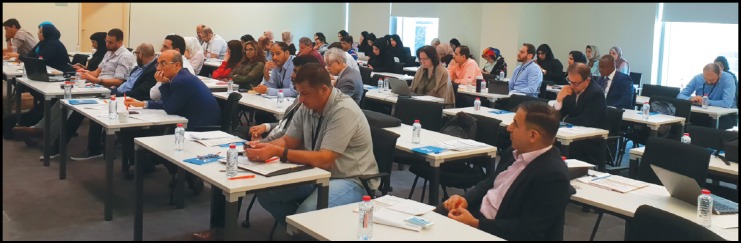
A view of the workshop participants.

Last presentation in this session was by **Dr. Ahmed Mandil** who talked about Web Medical Search Engines. It is used for literature search while writing papers to find out what has already been done and what information is already available on that particular topic locally, nationally, in the region and internationally. Some of the important Web based search engines, databases include PubMed, PubMed Central, Cochrane, Science Citations, scientific journals. National databases will include DHS, ENSTINET, IranMedex, SaudiMedLit, Islamic Science Citation Index (ISC), and CAPMAS. Etc. Cochrane is the single most reliable source for evidence on effects of healthcare. It brings together in one place research on effectiveness of different healthcare treatments, interventions and is considered as a gold standard in evidence based medicine. TRIPS is a clinical search engine designed to allow users to quickly and easily find and use high-quality research evidence to support their practice. He then showed different Web Based Medical Search Engines and Data Bases, explained how they work. He also referred to different databases, resources put up by WHO, age details about Global Observatory for eHealth, IRIS-WHO Digital Repository, WHO IMEMR one of the regional databases which has included 26% of the six hundred journals published from the EMRO Region. Various countries in the region has also established Natonal Databases while WHO website also contains country specific information related to health. For registration of clinical trials, there is website CliicalTrials.gov. Where one can search the desired information is based on the research question, he remarked

**Dr. Ahmed Mandil** was the first speaker in the next session who talked about writing Introduction. Some of the questions which one should ask oneself before writing this section in a scientific paper, he said, are what do I have to say? Is it worth saying? What is the right format of the message? Who is my audience? Which is the right Journal to convey this message? It is important to ensure that the introduction is interesting from the first sentence. Introduction, Dr. Ahmad Mandil stated must be concise yet informative. It includes what is already known in this field, reflects the importance of carrying out this study and its rationale, what is known and what is not known. At the end the objective of the study must be restated.

**Mr. Shaukat Ali Jawaid** discussed how to write Methods. This section, it was pointed out must answer the important questions i.e. how the study was designed? How it was carried out? How the data was analyzed? When and where the study was conducted? In case one wants to find an answer to a question, one must sate what hypothesis was being tested? An intervention should result in a particular effect i.e increase in survival or improvement in outcome. For example the study of two antibiotics might compare the cure rates. The null hypothesis in this situation will be that there is no difference with cure as the outcome variable. It is important to give exact test used to analyze the data statistically. If the test is standard include a reference otherwise give complete minute details including the computer software and version used. Keep description brief, show how randomization was done, how participants were recruited chosen, reasons for excluding participants, mention ethical features, Ethics Committee/IRB approval, actual details of materials used, exact drug dosage and exact form of treatment. If the study is complex a diagram may be helpful. Give details of what disease states have been excluded, how these were defined and diagnosed, what medication lead to exclusion from the study, alcohol and tobacco alter drug response. Exact form of treatment used should be described in a way which allows replication. Describe strain of animals in lab study, describe how solutions were prepared, methods which are uncommon must be described fully. Apparatus used must be described in sufficient detail to allow the reader to be confident of the results reported and ensure calibration of the instruments used. A good methods, he stated, should answer some questions. Text should describe the question being asked, what was being tested, how trustworthy are measurements. Were these measurements recorded, analyzed and interpreted correctly? Finally would a qualified reader be able to repeat the experiment in the same way, he added.

Results is about reporting of what did you find - Karen Shashok

**Ms. Karen Shashok** discussed presentation of Results. It should focus on questions asked in the introduciton. Figures and tables, she said, should focus on the question asked and the data should help the readers answer it for themselves. Make sure that there is no repetition of data in text, tables or figures. She laid emphasis that one should use simple language, introduce abbreviations correctly and then use them consistently. Tables and figures should have headings and footnotes respectively. Results is about reporting of what did you find? If need be one can take the help of graphic designers for drawing tables and figures.

**Dr. Hamid Yahya Hussain** described how to write Discussion in a scientific paper. One can start with major findings of the study, explain meaning of the findings and why the findings are important. Relate the findings to the similar studies. State clinical relevance of these findings, acknowledge the study limitations. Discussion is all about what the results mean and implications of the findings. Discussion, he further stated should include summary of major findings, what do the findings mean, are they consistent with previous studies and if not why, try to find out the reasons for that. Interprete findings, their implications, limitations. Summary of the discussion section is usually conclusions which should succinctly summarize implications of findings. Refrain from making sweeping statements or conclusions which are not supported by the study findings. It should have a take home message for the readers. Some journals have specific instructions how to write Discussion, hence in that case follow those instructions. Do not over inflate the importance of the findings. While comparing your results compare it with the studies done in the last five years. Use of too old references is considered a weakness of the study and the paper may be rejected. It is also extremely important to highlight the limitations of the study. This can be addressed by the investigators in future studies. Clinical relevance, importance and strength of the study should be highlighted. It should also be clear to whom your recommendations are directed and what should be done in future to change their practice. One cane come up with new policy or new interventions. It is also important not to criticize other people’s work but discuss it in a scientific way as to why there are differences. There can be differences in selection of patients or there could be cultural differences or difference in dietary patterns.

Conclusions should have a take home message for the readers - Dr. Hamid Yahya Hussain

His next presentation was on citing references. While citing references, the objective. Dr. Hamid Hussain said is to give credit to others work. One should identify the gap and fill that gaps. Websites of important institutions, technical reports by working groups are also important source of references apart from scientific journals and books. Follow the instructions of the journal to which you wish to submit your manuscript while writing references.

**Ms. Karen Shashok** described how to write an abstract and select a title. She laid emphasis that one should avoid using abbreviations in abstract and title unless it is essential. Structured abstract will vary depending on the type of the article. In original research it is usually in four sub-headings i.e objective, methods, results and conclusions with key words. Case reports may have a one paragraph summary, short communication and clinical case series can have structured abstract just like an original article. The title, she stated, should contain key information. A title reflects the contents i.e. participants, population, conditions where what setting, experimental or observational. Follow the word limit for abstracts set by the journal. One has to avoid abbreviations because all readers may not be specialist in your area hence they will not read it. In abstract one is just reporting and not trying to convince the readers. One may have to revise the abstract once the final manuscript has been prepared. There should be no discrepancy in figures in text and tables. Read the paper carefully, proof read before final submission. In short title Ms. Karen Shashok stated should be brief, informative, interesting and give main idea of the work performed.

Highly specialized manuscript should focus on small but specific audience-Phillip Dingwall

**Dr. Phillip Dingwall** discussed how to select a suitable journal. Some of the points which must be remembered, he said, include that it should be read by the peers, should ensure more citations while academic credibility of a good journal is also very important. Publication in a good journal will disseminate it to peers and support your academic career. Make sure that your paper matches the aim of the journal. If your paper is general and could also be read by non-technical audience, then consider submitting to a multidisciplinary journal. However, if your manuscript is highly specialized, then one should focus on small but specific audience. Reaching the right audience is more important than just any audience, he remarked. While selecting the journal other things which one should consider include is the journal easy to find by other researchers, does it has good visibility and readership, is it listed in important electronic databases, is the journal indexed and covered by important indexes, is it available online as well as in print. Does the journal has an Impact Factor and are the articles in the journal assigned Digital Object Identifier (DOI). One can also search that journal online to see if it does come among the top research results. Good journals have well represented established editorial boards membership which includes eminent medical personalities. Is the journal sponsored by an important institution or organization? Well regarded journals attract a large number of submissions and many such journals have an acceptance rate of just about 10-15%. Look at the journal’s peer review process, avoid and do not get trapped by Predatory Journals. Some journals have processing fee as well as publication charges. Open access journals, Dr. Phillip Dingwall stated operate under a number of financial models for authors.

Electronic Manuscript Management systems have been designed to make the publishing process more efficient, readily accessible to authors and reviewers from all over the world

This was followed an open house discussion wherein components of a scientific paper, important information which different sections of the paper i.e. Structured Abstract, Introduciton, Methods, Results, Discussion should include. In fact it was total revision of the day’s presentations

On Day-2 of the workshop **Mr. Shaukat Ali Jawaid** discussed the authorship criteria by International Committee of Medical Journal Editors (ICMJE), highlighted the background to the formation of ICMJE. Last time it was revised in 2015 when the fourth criteria was added which is as under:

Many publishers now use electronic manuscript submission and peer review systems to manage their publications hence authors must familiarize them with these

1. Substantial contribution to conception and design, acquisition of data, or analysis and interpretation of data
2. Drafting the article or revising it critically for important intellectual content.
3. Final approval of version to be published.
4. Agreement to be accountable for all aspects of the work in ensuring that questions related to accuracy or integrity of any part of the work are appropriately investigated and resolved.

There is no limit to the number of authors and all those who have made significant contributions to the study and deserve authorship can be listed. However, authorship is a constant problem faced by the editors, remains under discussion and no perfect solution has been found so far. It all depends on the Trust between the authors and the Editors. He also gave some examples wherein those who agreed to add their name as authors without knowing anything about the study, had to face serious repercussions in their professional career. Decision about authorship and the order of listing of authors should be decided before the start of the study to avoid any dispute later. Those who cannot be listed as authors but have helped in the study through different ways should be included in acknowledgment. Since acknowledgment also means endorsement, there is a debate to get a written consent from those who are being acknowledged by the authors.

Most journals send detailed guidelines to the reviewers with manuscript & this Performa also states what is expected from the Reviewers - Farhad Handjani

## Ghost authors

These, he said, are defined as “Someone who secretly does artistic or literary work for another person, the later taking the credit”. They are considered paid literary workers. Sometimes they are considered as a necessary help for busy physicians in preparing the manuscript, literary materials. Some feel that instead of condemning them, they should be encouraged but the Authors must acknowledge the assistance rendered by them

**Figure:**
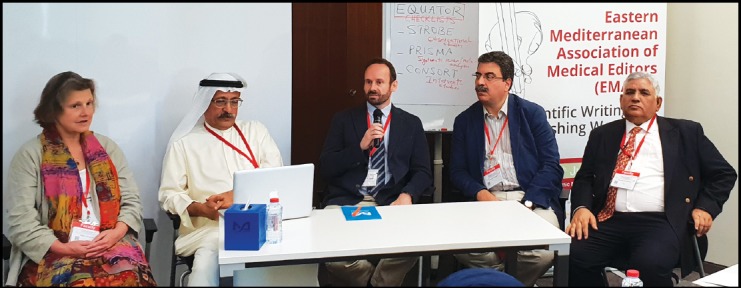
Ms. Karen, Prof. Yousef Al Bastaki, Phillip Dingwall, Prof. Farhad Handjani and Mr.Shaukat Ali Jawaid President and Secretary of EMAME respectively participating in the panel discussion during the workshop on Scientific Writing and Publishing held at Dubai from June 22-24, 2018.

## Gift Authorship

It is a menace and considered scientific misconduct. Most often it is the junior faculty members, postgraduates who add the name of their Head of the Dept., institution head just to please them. Gift authorship is an academic misconduct which promotes intellectual corruption

## Contributor ship

ICMJE authorship criteria, he said, is a recommendation, suggestion which is not mandatory. Even otherwise in the real world, most often those listed as authors cannot fully meet the above mentioned four criteria. Hence a new concept of contributor ship was coined in which the job done by each individual author in the study is mentioned separately. This is now being practiced by a large number of journals.

In case of a study by a Group, the main authors should be identified alongwith the name of the group and all others can be added as co-investigators at the end of the manuscript.

**Dr. Phillip** **Dingwall** then discussed the online submission and how it works. This presentation was prepared by **Prof. Waleed Al-Faisal** who unfortunately could not participate due to an accident. It was pointed out that many publishers now use electronic manuscript submission and peer review systems to manage their publications. Hence, it is important for the authors to get familiar with these as it could save valuable time and help them communicate better with the Editors. Most of the available programmes i.e. Scholar-One, Editorial Manager, and Open Journal System works the similar way. All these systems have been designed to make the publishing process more efficient, readily accessible to authors and reviewers from all over the world. He then discussed in detail the whole process on Scholar-One how it works. While submitting academic affiliations should be preferred by the authors.

Editors should become familiar with best practice in editing, peer review, research ethics and establish programme to monitor journal performance

**Prof. Farhad Handjani** made a presentation on Peer Review process and pointed out that it helps the editors to judge the suitability of the manuscript for publication. They also help improve the manuscript. Peer Review must be unbiased and a critical review. Feed back is important as it helps improve the manuscript. Reviewers learn all this by attending training courses, workshops while some learn it on the job. Most journals usually send the Reviewers Performa alongwith the manuscript to the reviewer which has detailed guidelines and what is expected from the Reviewers. One does not need to accept every invitation to review, if the manuscript is out of your area of expertise, one should decline and inform the editor. At times the reviewers might have to take help from other colleagues as well. To be a Reviewer for a good quality standard journal is an honour and privilege. Some journals give CME Credit points for promotion. He also referred to post publication reviews, wherein articles are published and authors can make changes in the paper later on in the light of the reviewer’s comments and suggestions. Single blind, double blind and open peer review system all have their advantages and disadvantages.

**Ms. Karen Shashok** stated that peer review is partly objective and partly subjective. It is a method to improve published articles. Major publishers, she pointed out, are enemy of Open Access Journals. Predatory journals do not use peer review. They are more interested in making money. Negative comments in peer review could be due to actual errors in research, manuscript. At times wrong files are submitted. Journal instruction for authors are not followed carefully. Open Peer Review system does improve the quality of review. Authors are supposed to give point wise response to the comments and suggestions while responding and all the changes made in the revised manuscript should be highlighted for easy identification which saves lot of time and the Editors can make a quick decision. The authors should not make the changes if they do not agree with the reviewer’s comments but they can challenge it with evidence. Reviewers are not always right. Peer Reviewers might provide some input but basically it is the job of the authors to do a good job, she remarked.

Editors must enjoy Editorial Freedom to determine editorial policy, choose reviewers, hire & dismiss the editorial staff, choose editorial writers

In the next session **Mr. Shaukat Ali Jawaid** talked about Rights and Responsibilities of the Editors. He pointed out that there is a delicate balance and connection between rights and responsibilities. Rights must be used responsibly particularly while dealing with others. Editors have responsibilities to readers to educate them by communicating clear and relevant information, explain the editorial policy, provide accurate information, encourage comments, discussion, provide clear instructions, explain editorial and peer review policy, ensure authors are treated respectfully, promptly respond to queries, ensure quick publication of manuscripts and sympathetic consideration of appeals.

Almost 80% research is supported by Pharma industry hence it is important that we work with them in close collaboration but uphold professional ethics-Yousef Al-Bastaki

Editor’s responsibilities to Reviewers include explaining the review process clearly, giving them sufficient time to review, provide them feedback. Editors have to respect not only authors, readers and reviews but human subjects as well. They must make the whole process transparent, protect confidentiality of human subjects and promote self correction by publishing retractions. They should also take responsibility for improving level of scientific investigations, ensure honesty and integrity, and manage conflict of interest and separate editorial and business functions of the journal. Editors should become familiar with best practice in editing, peer review, research ethics and establish programme to monitor journal performance. They also have responsibilities to public, science and advertisers i.e. providing clear health information, quick reporting of significant public health issues, promoting high quality science and educate the scientists. Advertising policy should be clear and ensure equal treatment to all advertisers.

Editors have some rights as well which include Editorial Freedom which is extremely important so that they can determine editorial policy, choose reviews, hire and dismiss the editorial staff, choose editorial writers and solicit manuscripts on controversial topics.

Predatory Journals mostly trap postgraduates, junior faculty members who are eager to get their manuscripts published immediately-Farhad Handjani

His next presentation was on How the Medial Editor office works. Editor, Mr. Shaukat Ali Jawaid stated is the Capitan of the Ship who should learn how to manage the challenges. He has to act as Gate Keeper, Manager, Leader, and Teacher. Editors must know the journal staff and how to make best use of their expertise. Editors do lot of teaching and training as well. Open Access is the way forward in scientific publishing but there is resistance to it in North America particularly from big publishes. At present there are different business models and the Editor has to select any one of them which ensures sustainability in the long run. Editorial staff and working of Medical Journal office will depend on the specialty, frequency of publication. It is important to acquire good Manuscript Management System, train authors and reviewers how to use it. Editorial Board should be broad-based, ensure to have atleast 25% of the members from overseas. Ownership of the journal also determines how it will function as sometime institutions and specialty organizations which own the journal interfere too much and seldom give editorial freedom to the editors. However it is important to avoid *“Show Pieces*” in the Editorial Board which should consist of those who are keen, have time and are prepared to work. Categorization of submitted manuscripts, Indexation of the Journal in various databases, how to generate resources, academic misconduct, ethical issues and editorial issues all have to be dealt with, hence the Editor must be competent, intelligent and experienced to handle all these issues satisfactorily with the help of his team.

**Figure:**
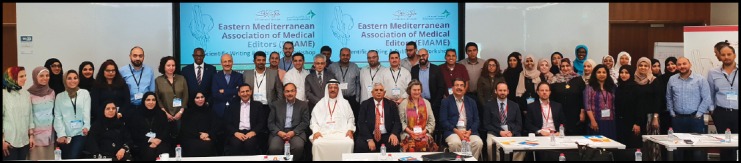
Eastern Mediterranean Association of Medical Editors (EMAME) in collaboration with Dubai Health Authority organized a three day workshop on Scientific Writing and Publishing at Dubai from June 22-24th, 2018. The group photograph taken on this occasion shows the facilitators of the workshop with the workshop participants.

## Hands on Exercises

This was followed by Hands on Exercises in small groups with facilitators. Mr. Shaukat Ali Jawaid had prepared two case scenarios regarding authorship which was discussed in detail and the participant were made aware of problems encountered and how they are resolved at times not to the entire satisfaction as junior researchers, postgraduates often have to work under very difficult circumstances and they cannot afford to annoy their seniors. Karen Shashok had prepared some Reviews and the comments were also discussed by the participants and the facilitators. The quality of the Reviews, the competence of the reviewers also came under discussion. Qualities of a good review were highlighted by the facilitators.

On last day of the conference **Mr. Phillip Purnell** from Knowledge-E talked about Scientometrics which means qualitative evaluation of impact based on citations, Impact Factor and rankings, Indexing. He also talked about the White Lists and Black Lists of Journals. It was Eugene Garfield who gave the concept of Impact Factor. He then described how IF is calculated. Drawbacks and limitations of IF and how some journals manipulate it also figured during the discussion. He also referred to the DORA Declaration which was highly critical of IF. He then briefly talked about H-index and how it is calculated. The impact of Negative Citations and Self Citations by the authors and Journal level besides limitations of using bibliometircs were also highlighted.

Scientometrics means qualitative evaluation of impact based on Citations, Impact Factor, rankings and Indexing - Phillip Purnell

**Dr. Ahmad Mandil** discussed Research Ethics and highlighted the importance of Informed Consent for conducting the study. Ethics, he opined, must be observed at all stages of research. Each medical institution should have a Research Ethics Committee. He also referred to publication ethics, authorship issues, conflict of interest, editorial freedom, redundant publications and simultaneous submission of manuscripts to different journals by the authors which is highly unethical. **Prof. Yousef Al-Bastaki** briefly talked about Ghost Authors. He was of the view that assistance in writing must be acknowledged. He then shared a few examples how pharma industry manipulates the results in clinical trials which they sponsor and they also retain the data in their control. However, 80% of research is supported by Pharma industry hence it is important that we work with them in close collaboration but uphold professional ethics, he remarked.

**Prof. Farhad Handjani** talking about Open Access Publishing gave details of different business models i.e. author pay model, readers pay model. Open Access varies in different countries. Production, Archiving, Website maintenance all cost money and journals have to generate funds for all this. He also referred to self-archives, institution repositories. Predatory journals have a very high acceptance rate and offer fast publication but they conceal their publication location, mostly operate on the net, hence it is important that one should not get trapped. They mostly trap the postgraduates and junior faculty members who are eager to get their manuscripts published immediately. He also highlighted reasons for rejection of manuscripts and mentioned that Journal Editors usually prefer to publish ground breaking new research. Some time the editors reject the manuscript through in-house initial screening and review and sometimes it is rejected after peer review. In some Journals it is the Editor and in some it is the Editorial Board which makes the final decision of rejecting a manuscript. Most common reason for rejection is poor quality of the research, Lack or originality and significance, mismatch with the journal. Space constraints, high volume of submissions, more submissions on a particular topic or discipline, inadequately prepared manuscript with poor presentation. Author’s failure to follow instruction, poor English and Grammar, flaws in study design, small sample size, poor literature search, and too old references, poorly designed tables, illustrations are some other reasons for rejection, he added.

**Figure:**
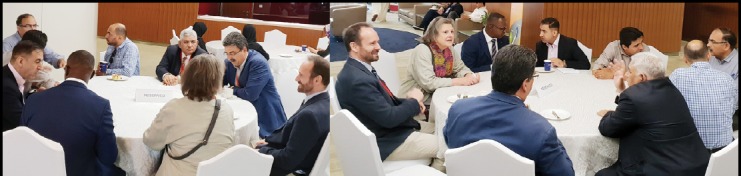
A view of the Hands on Exercise by the participants with the facilitators during the three day workshop on Scientific Writing and Publishing organized by Eastern Mediterranean Association of Medical Editors (EMAME) in collaboration with Dubai Health Authority at Dubai from June 22-24th, 2018.

In his concluding remarks **Prof. Yousef Al-Bastaki** said that he was impressed with the level of participation and the excellent contributions made by the faculty. We will be too glad to assist in collaborating in such academic activities in future as well to promote the art of medical writing and scientific publishing which will promote research culture and also improve the quality of manuscripts being published from the Region. He also thanked the University for their Support and hosting the workshop.

